# Bacterioferritin of Magnetospirillum gryphiswaldense Is a Heterotetraeicosameric Complex Composed of Functionally Distinct Subunits but Is Not Involved in Magnetite Biomineralization

**DOI:** 10.1128/mBio.02795-18

**Published:** 2019-05-21

**Authors:** René Uebe, Frederik Ahrens, Jörg Stang, Katharina Jäger, Lars H. Böttger, Christian Schmidt, Berthold F. Matzanke, Dirk Schüler

**Affiliations:** a Department of Biology, Chemistry and Geosciences, Universität Bayreuth, Bayreuth, Germany; b Isotopes Laboratory, Section Sciences, Universität zu Lübeck, Lübeck, Germany; University of California, Berkeley

**Keywords:** bacterioferritin, biomineralization, ferritin, magnetite, oxidative stress, prokaryotic organelle

## Abstract

The biomineralization pathway of magnetite in magnetotactic bacteria is still poorly understood and a matter of intense debates. In particular, the existence, nature, and location of possible mineral precursors of magnetite are not clear. One possible precursor has been suggested to be ferritin-bound ferrihydrite. To clarify its role for magnetite biomineralization, we analyzed and characterized ferritin-like proteins from the magnetotactic alphaproteobacterium Magnetospirillum gryphiswaldense MSR-1, employing genetic, biochemical, and spectroscopic techniques. Transmission Mössbauer spectroscopy of the wild type (WT) and a bacterioferritin (*bfr*) deletion strain uncovered that the presence of ferrihydrite in cells is coupled to the presence of Bfr. However, *bfr* and *dps* deletion mutants, encoding another ferritin-like protein, or even mutants with their codeletion had no impact on magnetite formation in MSR-1. Thus, ferritin-like proteins are not involved in magnetite biomineralization and Bfr-bound ferrihydrite is not a precursor of magnetite biosynthesis. Using transmission electron microscopy and bacterial two-hybrid and electrophoretic methods, we also show that MSR-1 Bfr is an atypical representative of the Bfr subfamily, as it forms tetraeicosameric complexes from two distinct subunits. Furthermore, our analyses revealed that these subunits are functionally divergent, with Bfr1 harboring a ferroxidase activity while only Bfr2 contributes to heme binding. Because of this functional differentiation and the poor formation of homooligomeric Bfr1 complexes, only heterooligomeric Bfr protects cells from oxidative stress *in vivo.* In summary, our results not only provide novel insights into the biomineralization of magnetite but also reveal the unique properties of so-far-uncharacterized heterooligomeric bacterioferritins.

## Abstract (precis)

Magnetotactic bacteria like Magnetospirillum gryphiswaldense are able to orient along magnetic field lines due to the intracellular formation of magnetite nanoparticles. Biomineralization of magnetite has been suggested to require a yet-unknown ferritin-like ferrihydrite component. Here, we report the identification of a bacterioferritin as the source of ferrihydrite in *M. gryphiswaldense* and show that, contrary to previous reports, bacterioferritin is not involved in magnetite biomineralization but required for oxidative stress resistance. Additionally, we show that bacterioferritin of *M. gryphiswaldense* is an unusual member of the bacterioferritin subfamily as it is composed of two functionally distinct subunits. Thus, our findings extend our understanding of the bacterioferritin subfamily and also solve a longstanding question about the magnetite biomineralization pathway.

## INTRODUCTION

Iron is essential for almost all organisms. Because of its versatile biochemical properties, it serves as a cofactor in a multitude of biochemical reactions, including respiration, photosynthesis, or DNA synthesis (1). However, excess iron can be toxic due to its ability to increase the formation of reactive oxygen species via the Fenton reaction (2). To avoid iron-mediated toxicity, bacteria use regulatory sensor proteins (e.g., ferric uptake regulator [Fur]) to determine intracellular iron concentrations and transcriptionally control genes involved in the import, efflux, and metabolism of iron (3). Additionally, cells employ proteins of the ferritin-like superfamily to store excess iron as an inert iron mineral phase and thus prevent Fenton chemistry (2). Ferritin-like proteins with iron storage capability are found in the ferritin (Ftn), heme-containing bacterioferritin (Bfr), archaeoferritin (Afr), and DNA-binding proteins from starved cells (Dps) or Dps-like subfamilies (4–6). All of these proteins share a four-helical bundle structural motif and are able to self-assemble into sphere-shaped hollow tetraeicosameric (Ftn, Bfr, and Afr) or dodecameric (Dps and Dps-like) nanocages that engulf an iron mineral core (4). Iron mineralization in the interior of the nanocages is catalyzed by a ferroxidase center (FC) which is formed by dinuclear metal ion binding sites in the center of each Ftn and Bfr subunit or at subunit interphases in Dps proteins (7, 8). Most iron-storing ferritin-like proteins form homooligomeric shells and thus have a fixed number of FCs per nanocage. Vertebrate ferritins, however, are heterooligomeric assemblies. In humans, for example, ferritin is composed of heavy (H)- and light (L)-chain ferritins which share only 55% sequence similarity and have different functionalities (9). While H-ferritin provides the FC residues, L-ferritin is thought to enhance oligomer stability and iron influx into the nanocage (10).

A rather unusual role of ferritin-like proteins was proposed for magnetotactic bacteria (MTB) (11). In *Magnetospirillum* species, a ferritin-like component was found to play a major role in the intracellular biomineralization pathway of membrane-enclosed ferrimagnetic magnetite [Fe^2+^(Fe^3+^)_2_O_4_] crystals, called magnetosomes, which enable magnetospirilla and other MTB to orient themselves along the Earth’s magnetic field lines to find growth-favoring anoxic or microoxic zones within their aquatic habitats. Early Mössbauer spectroscopic and electron microscopic analyses indicated that crystallization of magnetite in Magnetospirillum magnetotacticum proceeds via precursors, including a ferritin-like ferrihydrite component (12). Using X-ray absorption near-edge structure (XANES) and high-resolution transmission electron microscopy (TEM), Fdez-Gubieda et al. (13) reported that magnetite is also rapidly biomineralized from a ferritin-like ferrihydrite precursor in Magnetospirillum gryphiswaldense MSR-1. An investigation on *M. magneticum* revealed the presence of a highly disordered and phosphate-rich ferric hydroxide phase consistent with prokaryotic ferritins outside the magnetosome vesicle (14). Interestingly, in this study the authors could show that this iron mineral phase transforms via purely inorganic ferrihydrite-like nanometric ferric(oxyhydr)oxides to magnetite within the magnetosomes (14). Based on Mössbauer spectroscopic analyses of MSR-1, however, a magnetite biomineralization model without mineral precursor phases was suggested (15). Nevertheless, it was proposed that a ferritin-like compound is required for the release of iron at the magnetosome-compartment interface (15). Preliminary biochemical analyses provided evidence that the ferritin-like ferrihydrite metabolite is of proteinaceous and not inorganic origin (15). The authors could enrich a reddish, high-molecular-mass protein (*M* > 100 kDa) that carried large amounts of iron. However, the ferritin-like component has not been identified at the molecular level yet.

In order to identify the ferritin-like iron metabolite and clarify its role for magnetite biomineralization, we started to analyze ferritin-like proteins of MSR-1. Using genetic, biochemical, and spectroscopic techniques, we show that, contrary to previous assumptions, ferritin-like proteins are not required for magnetite biomineralization but involved in the resistance to oxidative stress. We also provide evidence that Bfr of MSR-1 is a heterotetraeicosameric protein complex consisting of subunits with distinct biochemical and catalytic properties. Furthermore, we show that the Bfr1 subunit requires interaction with Bfr2 to form stable nanocages.

## RESULTS

**FIG 1:**
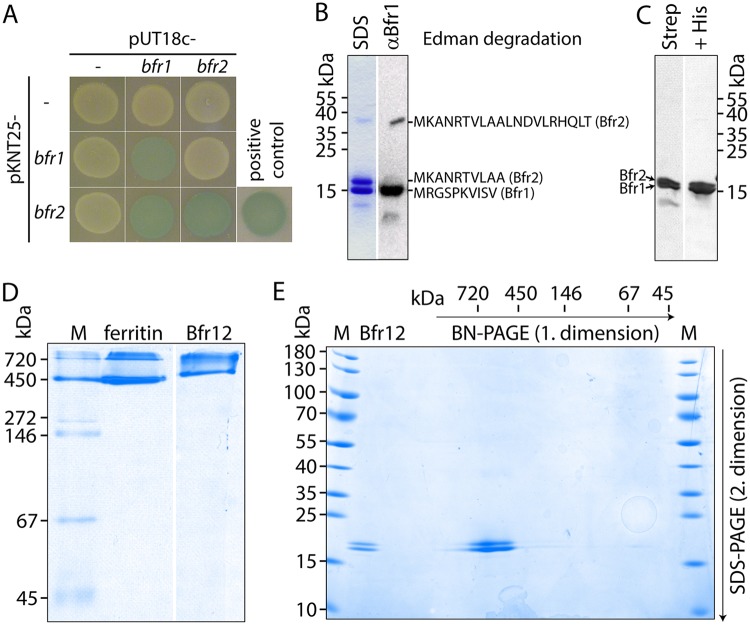
Bfr of MSR-1 is a heterotetraeicosamer. (A) Detection of Bfr protein (self-)interactions via bacterial two-hybrid analysis. Five-microliter cell suspensions of E. coli BTH101 coexpressing T18 and T25 fusions of *bfr1* and *bfr2* as well as control strains with empty vectors were grown on LB–X-Gal–IPTG medium at 30°C for 20 h. Positive control, E. coli BTH101 cotransformed with pKT25-*zip* and pUT18c-*zip*. (B) Coomassie blue-stained SDS-PAGE (13 to 20%) and anti-Bfr1 Western immunoblot analysis of 4 μg SEC- and AEC-purified, untagged MSR-1 Bfr after overexpression from pET-15b-Bfr in E. coli BL21-gold. Sequences retrieved from Edman degradation of the corresponding bands are indicated. (C) Coomassie blue-stained SDS-PAGE (15%) analysis of 4 μg Strep-tag affinity (left) and Strep-tag plus Ni-NTA-affinity (right)-purified MSR-1 Bfr after overexpression from pET-51b-Bfr12 in E. coli Rosetta(DE3)pLys. (D) Coomassie blue-stained 4 to 12% BN-PAGE gel of 6 μg horse spleen ferritin and 8 μg tandem-affinity-purified MSR-1 Bfr. M, marker (native marker liquid mix for BN/CN [Serva]). (E) Coomassie blue-stained 2D-BN/SDS-PAGE gel of Strep-tag affinity-purified MSR-1 Bfr after overexpression from pET-51b-Bfr12 in E. coli Rosetta(DE3)pLys. The arrow indicates the position and direction of the Bfr-containing BN-PAGE gel slice from the first dimension. Bfr12, 1 μg of SDS-treated MSR-1 Bfr loaded directly on the SDS-PAGE gel (without previous BN-PAGE). M, marker (prestained PageRuler protein ladder [Thermo Scientific]).

Eleven genes encoding proteins with at least partial homology to eight different ferritin-like subfamilies were identified in the genome of MSR-1 (16). However, only three proteins (MSR1_27050 [Bfr1], MSR1_27040 [Bfr2] [bacterioferritins], and MSR1_39700 [Dps]) belonged to subfamilies with the capability to store iron (see Fig. S1A in the supplemental material). Among these, Bfr1 and Bfr2 first caught our attention since both proteins are encoded by genes that are separated by only 11 nt and seem to form a bicistronic operon (Fig. S1B). Furthermore, sequence analyses revealed the lack of a Met residue at position 52 in Bfr1 which is essential for axial heme binding at subunit interfaces (17). Thus, Bfr1 is likely unable to contribute to heme binding. In contrast, several residues of the FC are mutated in Bfr2 (Fig. S1C). These findings suggested that the Bfr proteins of MSR-1 are functionally divergent and might form a heterooligomeric bacterioferritin complex. To test if Bfr1 and Bfr2 indeed interact, a bacterial two-hybrid assay was performed. Therefore, *bfr1* and *bfr2* were cloned in frame with either the N-terminal T25 domain or the C-terminal T18 domain of the Bordetella pertussis CyaA adenylate cyclase, respectively. The resulting T18 and T25 plasmids were cotransformed into Escherichia coli BTH101 in different combinations with empty pUT18c and pKNT25 vectors as controls. Protein-protein interactions were evaluated after growth of spotted cell suspensions on LB–X-Gal–IPTG plates. While all control strain colonies remained white, strains coexpressing T18-*bfr1* and *bfr1-*T25 or T18-*bfr2* and *bfr2-*T25 formed dark blue colonies, indicating strong self-interactions (Fig. 1A). Interestingly, we also observed a strong interaction in strains coexpressing T18*-bfr1* and *bfr2-*T25, indicating a Bfr heterooligomerization. In contrast, strains coexpressing T18-*bfr2* and *bfr1*-T25 revealed no interaction, which, however, might have been caused by sterical hindrance of the T-domains in a heterooligomeric Bfr complex. To confirm the formation of heterooligomeric nanocages *in vivo*, we next aimed to purify the native protein complex from MSR-1. Unfortunately, various attempts employing classical (heat treatment, butanol extraction, and anion-exchange and size exclusion chromatography [AEC and SEC, respectively]) or recombinant (affinity purification of tagged Bfr) methods were not successful due to the low abundance of the protein, its temperature sensitivity, or its strong tendency to aggregate. Hence, we coexpressed *bfr1* and *bfr2* in E. coli. After purification of Bfr by SEC and AEC, four bands were observed on SDS-PAGE: two intense bands at approximately 15 kDa and 18 kDa and two faint bands of 12 and 36 kDa, respectively (Fig. 1B). The small 12-kDa band was attributed to a degradation product because its concentration increased concomitantly to the length of the expression period and was recognized by an anti-Bfr1 antibody. Edman sequencing of the 15-kDa band revealed sequences perfectly matching the N terminus of Bfr1, whereas the sequence from the 18-kDa band was identical to the first 10 aa of Bfr2. For the band at 36 kDa, a 20-aa-long sequence that had 100% identity to Bfr2 could be determined. In contrast to the band at 18 kDa, the 36-kDa band was also stained in immunoblots with anti-Bfr1 antibodies (Fig. 1B), further indicating formation of a heterooligomeric Bfr. However, the presence of two different homooligomers of the same molecular weight (MW) could still not be excluded using SEC and AEC. Therefore, we changed our strategy and used Strep tag affinity purification to isolate Strep-tagged Bfr1. By this method, Bfr2 could be copurified and was present at a similar ratio as Bfr1 as observed with SEC/AEC-purified untagged Bfr (Fig. 1C). Subsequent purification of His-tagged Bfr2 using Ni-NTA affinity chromatography resulted in the removal of the ∼12-kDa degradation product, whereas Bfr1 and Bfr2 were still present at similar ratios, indicating that the two proteins were present in the same complex (Fig. 1C). Next, purified Bfr extracts were loaded onto a blue native (BN)-PAGE gel to determine the MW of the complex. This analysis revealed the presence of two distinct bands of ∼500 kDa and ∼740 kDa, respectively (Fig. 1D). While these results suggested the presence of two different Bfr complexes, 2D-BN/SDS-PAGE analyses revealed that Bfr1 and Bfr2 were present in both high-MW bands (Fig. 1E). Since it is well known that vertebrate ferritins can form dimers (18) and horse spleen ferritin was also separated into a 450-kDa monomeric nanocage band and a 720-kDa dimeric nanocage band (Fig. 1D), we assume that the 740-kDa band of MSR-1 Bfr also reflects a dimeric Bfr nanocage fraction. Subsequently, we used transmission electron microscopy (TEM) to estimate the number of subunits of a single Bfr nanocage. The analysis of negatively stained protein complexes revealed an average outer diameter of 11.87 ± 1.02 nm (Fig. S2). Almost-identical complex sizes of 11.95 ± 0.76 nm were observed with tetraeicosameric horse spleen ferritin, which suggested that also Bfr of MSR-1 is composed of 24 subunits.

10.1128/mBio.02795-18.2 FIG S1 (A) Phylogenetic tree of ferritin-like proteins. The scale bar indicates an evolutionary distance of 0.5 amino acid substitution per site. Bfr1 proteins are labeled in red and Bfr2 sequences in light blue letters. Green, sequences of organisms with two or more Bfrs but no Bfr1 homologues. (B) Genomic organization of *bfr1* and *bfr2* genes. (C) ClustalW sequence alignment between Bfr1, Bfr2 from MSR-1, and Bfr from E. coli with schematic representation of Bfr_Ec secondary structure. Conserved residues are shown in black, and homologous residues are in gray. Black boxes above sequences indicate residues involved in heme binding, and white boxes indicate residues of the ferroxidase center. Download FIG S1, TIF file, 2.4 MB. Copyright © 2019 Uebe et al. 2019 Uebe et al. This content is distributed under the terms of the Creative Commons Attribution 4.0 International license.

10.1128/mBio.02795-18.3 FIG S2 Transmission electron micrographs of negatively stained Bfr12, Bfr1, Bfr2, horse spleen ferritin, and buffer samples. Download FIG S2, TIF file, 2.4 MB. Copyright © 2019 Uebe et al. 2019 Uebe et al. This content is distributed under the terms of the Creative Commons Attribution 4.0 International license.

**FIG 2:**
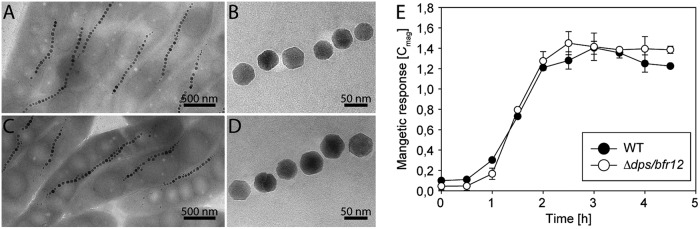
Bfr and Dps are not required for magnetosome formation in MSR-1. (A to D) Representative TEM micrographs of cells and magnetosome particles from MSR-1 WT (A and B) and Δ*dps/bfr12* (C and D) after growth under anaerobic conditions. (E) Time course of increase in magnetic response of WT and Δ*dps/bfr12* cultures after induction of magnetite biomineralization by addition of 50 μM iron citrate. Cells were passaged in the absence of iron three times before induction. The assay was performed in triplicate, and values are expressed as means, with standard deviations displayed as error bars.

Next, we were interested in the role of iron-storing ferritins for magnetite biomineralization and generated unmarked deletion mutants of *dps* and the *bfr12* operon. TEM analyses showed that both mutants formed magnetite crystals with an average diameter of 33 ± 9.7 nm (Δ*bfr12*) or 32.4 ± 8.3 nm (Δ*dps*) that were essentially identical to the WT (34.8 ± 10.2 nm). Also, the average numbers of magnetosomes (Δ*bfr12 *=* *33.1 ± 8.5 cell^−1^; Δ*dps* = 34.1 ± 11.1 cell^−1^) were similar to the WT 34.3 ± 8.4 cell^−1^ (Fig. S3), indicating that in both mutants magnetosome formation was not affected. To exclude the possibility that Dps and Bfr complement each other in terms of magnetite biomineralization, we aimed to delete *bfr12* in the *dps* mutant background. Under microaerobic conditions (2% O_2_), only a few clones that still contained the *bfr* genes were obtained. However, under strictly anoxic conditions we succeeded in the generation of the *dps/bfr12* double deletion mutant, suggesting that growth of the *dps/bfr12* mutant might be inhibited by oxygen. Thus, we analyzed magnetosome formation of the *dps/bfr12* mutant after growth under anoxic conditions. Unexpectedly, we failed to detect any significant difference from the WT with respect to magnetic response (WT = 1.42 ± 0.01 versus *Δdps/bfr12 *=* *1.4 ± 0.03), magnetosome numbers (WT = 28.2 ± 8.0 cell^−1^ versus Δ*dps/bfr12 *=* *28.8 ± 8.1 cell^−1^) as well as magnetite crystal diameters (WT = 41.0 ± 11.4 nm versus Δ*dps/bfr12 *=* *39.6 ± 12.0 nm) or crystal shape (Fig. 2A to D). Also, magnetosome induction experiments by addition of iron to cells grown under iron-limiting conditions revealed no differences between the WT and Δ*dps/bfr12* strains. In both strains, the magnetic response started to increase already 30 min after iron induction (Fig. 2E) and reached saturation 2 h postinduction, indicating that the ferritin-like proteins Dps and Bfr are not required for magnetite formation in MSR-1.

10.1128/mBio.02795-18.4 FIG S3 Representative TEM micrographs of MSR-1 WT, *Δdps*, and *Δbfr12* strains. Insets show magnification of the boxed areas. Download FIG S3, TIF file, 2.1 MB. Copyright © 2019 Uebe et al. 2019 Uebe et al. This content is distributed under the terms of the Creative Commons Attribution 4.0 International license.

**FIG 3:**
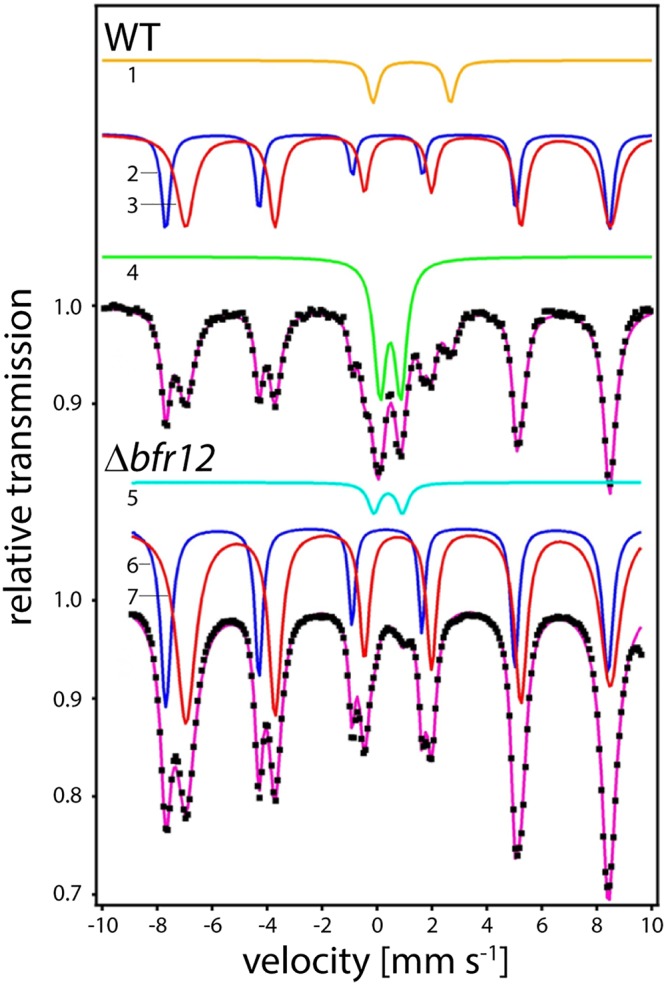
Mössbauer analysis of MSR-1 WT (upper part) and *Δbfr12* (lower part) whole cells. Transmission spectra (purple lines with black dots) were recorded at 130 K. Fitted subspectra of different metabolites are indicated by numbers (1 to 7). Similar metabolites are indicated by similar colors (yellow, blue, turquoise, red, and green, respectively). For details and parameters used to fit metabolite spectra, see Table 1.

Previous studies suggested that a bacterioferritin-like protein corresponds to the conspicuous ferritin-like iron metabolite implicated in magnetosome formation (12–15). To test this assumption, the WT and Δ*bfr12* strains were grown in the presence of 40 μM ^57^Fe(citrate)_2_, harvested in late logarithmic growth phase, and subjected to whole-cell transmission Mössbauer spectroscopic (TMS) analysis at 130 K (above the Verwey temperature of magnetite [*T_V_* = 113 K]). In spectra of the WT, three iron metabolites could be detected based on their Mössbauer parameters (isomer shift [δ], quadrupole splitting] Δ*E_Q_*], and hyperfine field [Bhf]) (Table 1, subspectra 1 to 4). The major component exhibits two magnetically split sextets typical of magnetite sites A and B (Fig. 3, s2 and s3, respectively). The second component was attributed to a ferrihydrite-like ferric high-spin species (s4) which is a marker for ferritin-like species. Evidence for this classification follows from a comparison of Mössbauer spectra from MSR-1 WT and the magnetite-free Δ*mamM* mutant measured at 130 and 4.2 K (19) (Table 1, s8 to s11). A minor doublet in the WT spectrum was attributed to a ferrous high-spin iron [Fe^2+^O_6_(X_6_)*^n^*]*^n^*^−10^ metabolite (Fig. 3, s1). In Δ*bfr12* cells, again, magnetite was identified as the main iron metabolite (Fig. 3, s6 and s7). In addition, a minor doublet component (Fig. 3, s5) belongs to a [Fe_4_-S_4_]^2+^ protein (19, 20), which is masked or not present in the WT. However, no ferrihydrite-like component (i.e., ferric high-spin species) was traceable in TMS spectra of the Δ*bfr12* mutant. These Mössbauer experiments substantiate that the ferrihydrite signal is caused by the iron mineral core of Bfr and that biosynthesis of magnetite does not require Bfr.

**TABLE 1:** Analysis of Mössbauer spectra^*a*^

Sample (whole cells) and subspectrum	Temp (K)	Metabolite	δ (mm s^−1^)	Δ*E_Q_* (mm s^−1^)	Γ (mm s^−1^)	Bhf (T)	Relative area (%)
WT							
1^*c*^	130	[Fe^2+^O_6_(X_6_)*^n^*]*^n^*^−10^	1.26	2.82	0.43		6.1
2	130	Magnetite A	0.38	0	0.3	49.5	23.7
3	130	Magnetite B	0.76	0	0.52	47.5	47.5
4	130	Ferritin-like	0.48	0.75	0.52		22.7
Δ*bfr12*							
5	130	[Fe_4_-S_4_]^2+^	0.40	1.04	0.50		3.0
6	130	Magnetite A	0.36	0	0.34	49.3	32.3
7	130	Magnetite B	0.76	0	0.55	47.4	64.7
WT^*d*^							
8	130	Ferritin-like	0.45	0.67	0.44		30.36
9	4.2	Ferritin-like	0.49	0.00	0.55^*b*^	49.5	26.5
Δ*mamM*^*d*^							
10	77	Ferritin-like	0.45	0.75	0.45		61.0
11	4.2	Ferritin-like	0.48	0.00	0.58^*b*^	47.83	61.8

a Summary of parameters used to fit the spectra of Fig. 3 and of spectra not shown in this contribution.

b Line with largest full width at half maximum.

c A ferrous iron metabolite with similar Mössbauer characteristics has been observed in many bacterial and fungal systems and was identified as cytoplasmic oligomeric ferrous sugar phosphate in E. coli (39).

d Data taken from reference 40: spectra document superparamagnetic transitions between 77 and 4.2 K, yielding complete magnetically split species indicating iron mineral cores as in mammalian ferritins.

**FIG 4:**
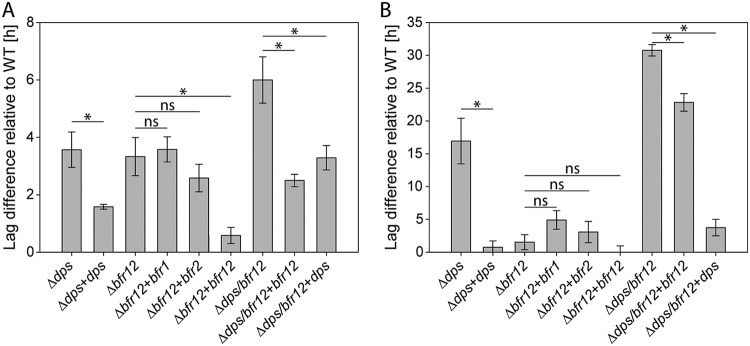
Bfr and Dps protect MSR-1 from oxidative stress. (A) Growth inhibition of MSR-1 strains by oxic conditions (21% O_2_). (B) Growth inhibition of MSR-1 strains by oxic conditions (21% O_2_) in the presence of H_2_O_2_. Growth inhibition is expressed as lag-phase duration difference of ferritin-like mutants and transcomplementation strains relative to the WT. Lag-phase duration difference was calculated by measuring the time to grow to an OD_565_ of 0.1 for each strain and subtracting the time that the WT required to grow to the same OD_565_. Values are given as means from at least 4 independent replicates ± standard error of the mean. *, *P* < 0.05 in two-tailed *t* test; ns, no significant difference (*P* ≥ 0.05 in two-tailed *t* test).

**FIG 5:**
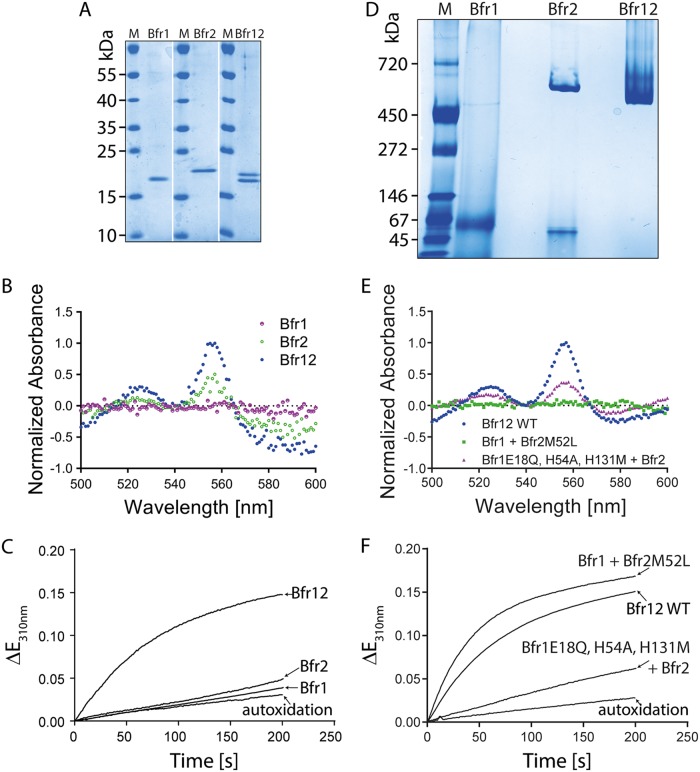
MSR-1 Bfr is composed of functionally distinct subunits. (A) Coomassie blue-stained 8 to 22.5% SDS-PAGE gel with Strep-tag affinity-purified Bfr1 (1 μg), Bfr2 (1 μg), and Bfr12 (2 μg). M, marker (prestained PageRuler protein ladder [Thermo Scientific]). (B) Pyridine hemochrome difference spectra of 50 μM purified Bfr1, Bfr2, and Bfr12. (C) Absorbance changes measured at 310 nm after the addition of 150 μM Fe(II)Cl_2_ to 1 μM Bfr1, Bfr2, and Bfr12, respectively. The autoxidation control contained an equal volume of buffer A1 instead of protein solution. (D) Coomassie blue-stained 6 to 15% BN-PAGE gel loaded with 18 μg Bfr1, 13.5 μg Bfr2, and 13.5 μg Bfr12, respectively. M, marker (native marker liquid mix for BN/CN [Serva]). (E) Pyridine hemochrome difference spectra of 50 μM purified Bfr12, Bfr1 plus Bfr2M52L, and Bfr1E18Q, H54A, H131M plus Bfr2. (F) Absorbance changes measured at 310 nm after the addition of 150 μM Fe(II)Cl_2_ to 1 μM Bfr12, Bfr1 plus Bfr2M52L, and Bfr1E18Q, H54A, H131M plus Bfr2, respectively. The autoxidation control contained an equal volume of buffer A1 instead of protein solution.

Since the *dps/bfr12* double deletion mutant could be generated only under anaerobic conditions, we suggested that Bfr might be involved in the resistance to oxidative stress. To confirm this assumption, we tested MSR-1 WT, Δ*dps*, Δ*bfr12*, Δ*dps/bfr12*, and corresponding transcomplemented mutant strains for susceptibility to oxidative stress (Fig. 4A and B). Under oxic conditions (21% O_2_), the mutant Δ*dps* (+3.6 ± 0.6 h; two-tailed *t* test, *P* value = 0.0039), Δ*bfr12* (+3.3 ± 0.7 h, *P* value = 0.0054), and Δ*dps/bfr12* (*+*6.0 ± 0.8 h, *P* value = 0.0002) strains showed significantly prolonged lag phases compared to the WT (20.9 ± 0.3 h). Transcomplementation of the mutant strains with *dps* or *bfr12* partially restored the growth delay. However, lag phases of the Δ*bfr12+bfr1* and Δ*bfr12+bfr2* transcomplementation strains were almost similar to that of the noncomplemented Δ*bfr12* mutant. In the presence of O_2_ and H_2_O_2_, the duration of the lag phase of the WT increased to 24.7 ± 0.6 h. Whereas the lag phase duration of the Δ*bfr12* mutant was also moderately increased, strains with a *dps* deletion showed strikingly prolonged lag phases relative to the WT (Δ*dps* = +16.9 ± 3.5 h; Δ*dps/bfr12* = +30.8 ± 0.9 h). Transcomplementation of Δ*dps* and Δ*dps/bfr12* strains with *dps* restored lag-phase duration to WT or Δ*bfr12* levels, respectively. In contrast, transcomplementation of the Δ*dps/bfr12* mutant with *bfr12* had a much weaker effect and reduced the lag phase by only 8 h to +22.8 ± 1.3 h relative to the WT. While these results confirmed our suggestion that Bfr is involved in the resistance to oxidative stress, it remained unclear why the Bfr1 subunit, containing the FC but lacking heme binding residues, was not able to complement the *bfr12* mutant. Previous studies showed that the catalytic activity of Bfr does not depend on heme but requires the FC (17, 21). To analyze Bfr1 in more detail, we overexpressed single *bfr* genes and *bfr12* in E. coli. After cell harvest, we already noted differences in the color of the cell pellets of the expression strains: while cell pellets of strains expressing *bfr2* or *bfr12* had a slightly reddish color, *bfr1* expression strains formed beige cell pellets (Fig. S4). We speculated that this difference is related to the heme-binding capability of the expressed subunit(s). Thus, after affinity purification we employed a pyridine hemochrome assay to determine the heme content of Bfr and the single subunits (Fig. 5A and B). Difference spectra of reduced and oxidized Bfr12 yielded a peak at 557 nm which indicated the presence of heme *b* (22), if also in very small amounts (0.24 to 0.6 heme *b* molecules per tetraeicosamer). Slightly lower heme contents were observed with Bfr2 (∼0.12 molecules per tetraeicosamer), while no heme could be detected in Bfr1 samples, confirming our initial assumption that Bfr1 is unable to bind heme. Next, the catalytic activities of the bacterioferritins were determined. The heterooligomeric Bfr12 showed the highest activity, whereas both single Bfr subunits revealed iron oxidation rates that were only slightly above the autoxidation control (Fig. 5C). While the low catalytic activities of Bfr1 or Bfr2 explain why they were unable to restore the lag-phase duration of the Δ*bfr12* mutant under aerobic conditions, it remained unclear why Bfr1 had such low activities. We thus subjected the single subunits and Bfr12 to BN-PAGE to analyze their oligomeric states. These experiments revealed that Bfr12 (500 and 740 kDa) and Bfr2 (550 kDa) mainly formed high-MW oligomers whereas in the Bfr1 sample only a minor fraction of higher oligomers (500 kDa) and a large fraction of ∼58 kDa (corresponding to trimers) was observed (Fig. 5D). Consistent with these observations, Bfr2 homooligomeric nanocages with a diameter of 12.16 ± 0.76 nm could be readily observed by TEM while Bfr1 samples contained no or only few nanocage-like structures with a diameter of 13.09 ± 0.84 nm (Fig. S2). These results indicate that Bfr1, in the absence of Bfr2, fails to efficiently assemble into a functional Bfr complex and explains why expression of *bfr1* alone was unable to complement the *Δbfr12* mutant. To finally prove that Bfr1 and Bfr2 have distinct functions and that the catalytic activity of Bfr12 is based on the FC of Bfr1, we analyzed the ferroxidase activity of a Bfr12 version in which the FC mutations of Bfr2 were introduced into Bfr1 (E18Q, H54A, and H131M). As expected, FC-negative Bfr12 showed drastically reduced iron oxidation rates compared to WT Bfr12 and heme-free Bfr12(M52L) (Fig. 5E and F).

10.1128/mBio.02795-18.5 FIG S4 Representative pictures of E. coli Rosetta(DE3)pLys cell pellets after overexpression of *bfr1*, *bfr2*, and *bfr12* (from left to the right), respectively. Download FIG S4, TIF file, 1.5 MB. Copyright © 2019 Uebe et al. 2019 Uebe et al. This content is distributed under the terms of the Creative Commons Attribution 4.0 International license.

## DISCUSSION

In this study, we identified and characterized ferritin-like proteins from MSR-1 to analyze their role during magnetite biomineralization. In contrast to previous reports, which proposed a central role for a ferritin-like component in the magnetite biomineralization pathway (12–15), we were unable to detect any defects or alterations during magnetite biomineralization in the Δ*dps*, Δ*bfr12*, and Δ*dps/bfr12* ferritin-like mutants. Thus, neither bacterioferritin nor Dps are required for magnetite formation in MSR-1. While it might be possible that other, so-far-uncharacterized, ferritin-like proteins encoded in the genome of MSR-1 are required for magnetite biomineralization, our data indicate that Bfr is identical to the ferritin-like component identified in earlier studies, since ferritin-like signals were absent from the Δ*bfr12* strain. Therefore, we propose that magnetite biomineralization in MSR-1 is independent of ferritin-like proteins. Furthermore, our results suggest that previous studies, which implicated ferritin-like ferrihydrite in the magnetite biomineralization pathway, simply failed to spatially resolve the independent biomineralization reactions within bacterioferritin and magnetosomes.

Unlike most bacteria with characterized bacterioferritins, MSR-1 contains two distinct *bfr* genes (*bfr1* and *bfr2*) organized in a putative bicistronic operon. Similar organizations have so far been found only in Neisseria gonorrhoeae (23) and *M. magnetotacticum* (24), while distinct *bfr* genes of cyanobacteria (25) and fluorescent pseudomonads (26) are dispersed in their genomes. Although these studies already suggested the existence of heterooligomeric nanocages, direct evidence that isolated Bfr proteins were located within the same protein complex, and did not represent two different homooligomers, was lacking (23–26). In contrast, using SDS-PAGE analyses of tandem-affinity-purified Bfr, bacterial two-hybrid analysis, and (2D)-BN-PAGE as well as TEM imaging, we could for the first time unequivocally show the formation of a heterooligomeric bacterioferritin consisting of 24 subunits.

Consistent with its phylogenetic affiliation with the Bfr subfamily, heme *b* was detected in purified Bfr, although at a very low concentration. While similarly low heme contents have been described for Bfr from E. coli upon unbalanced overexpression (27), we could show that Bfr1, in contrast to Bfr2, is unable to contribute to heme binding, most likely due to the lack of Met52, which is required for axial heme binding between the subunits (17). According to the weaker Bfr2 band intensities relative to those of Bfr1 in SDS-PAGE and 2D-BN-PAGE experiments, we suggest that heterotetraeicosameric nanocages have an unequal Bfr1:Bfr2 subunit stoichiometry of 14:10. Thus, MSR-1 Bfr may contain at maximum five heme *b* binding sites per nanocage, which corresponds to only ∼60% of the heme-binding capacity of Bfr from E. coli and most likely also contributes to the low heme content of isolated Bfr. Considering the proposed role of heme in facilitating the release of iron from the mineral core (21), and the increased iron content of heme-free compared to heme-containing Bfr in E. coli (17), heterooligomeric Bfrs with only a few heme-binding sites could have evolved to store increased amounts of iron. This might be supported by the fact that heteroligomeric Bfrs have mainly been proposed for organisms with relatively high iron demand (e.g., magnetotactic, photosynthetic, or pathogenic bacteria) (23–25). Furthermore, the reduced number of FCs per oligomer may also contribute to an increased iron storage capability since L-rich ferritins from liver or spleen have been found to incorporate much more iron than H-rich ferritins from heart muscle or brain cells (28).

In contrast to heme binding, residues essential for the ferroxidase activity are conserved in Bfr1 only, since in Bfr2 several ferroxidase site residues are mutated. However, despite the presence of an FC, Bfr1 extracts showed unexpectedly low ferroxidase activities. We suggest that the low catalytic activity of Bfr1 is mainly based on the small amount of Bfr1 homooligomers. This would also be consistent with the finding that expression of *bfr1* alone could not restore the growth of the Δ*bfr12* mutant under aerobic conditions. Interestingly, also other organisms, for which a heterooligomeric Bfr has been proposed, seem to require both subunits to assemble functional Bfr complexes. For example, in *Synechocystis* sp. strain PCC 6803, it was observed that upon deletion of *bfrB* (corresponding to Bfr2 from MSR-1), no oligomeric complexes composed of BfrA (corresponding to Bfr1 of MSR-1) could be isolated (25). Similarly, despite the presence of *bfrA*, no iron-containing oligomers could be detected in N. gonorrhoeae Δ*bfrB* mutants (23). While the structural basis of the poor Bfr1 oligomerization remains to be elucidated, sequence analyses suggest that several exchanges of residues critical for oligomerization of E. coli Bfr (K33E, Y45E, D56G, E60N, and E128Q) (29) prevent proper assembly.

With Bfr2, we obtained almost equally low ferroxidase activities as observed with Bfr1. However, in contrast to Bfr1, the majority of Bfr2 was found to be assembled as an oligomer. Thus, the low ferroxidase activity can be directly related to the absence of the FC. This idea is also supported by the low activity of Bfr12 in which the Bfr2 FC mutations were introduced into Bfr1(E18Q, H54A, and H131M). Thus, the high activity of the heterooligomeric MSR-1 Bfr is mainly caused by the FC of properly assembled Bfr1.

In summary, we showed that in contrast to several previous reports the ferritin-like proteins Bfr and Dps are not involved in magnetite biomineralization. Additionally, we have characterized a heterotetraeicosameric bacterioferritin with, similar to vertebrate ferritins, functionally distinct subunits.

## MATERIALS AND METHODS

### Strains, plasmids, and growth conditions.

Bacterial strains and plasmids are described in Text S1 in the supplemental material. E. coli strains were grown in lysogeny broth (LB) supplemented with kanamycin (25 μg ml^−1^) or ampicillin (50 μg ml^−1^) at 37°C with vigorous shaking (200 rpm). For cultivation of E. coli BW29427, LB was supplemented with dl-α,ε-diaminopimelic acid (1 mM). All MSR-1 strains were grown at 28°C or 30°C in modified flask standard medium (FSM) with moderate agitation (120 rpm) under aerobic, microaerobic, or anaerobic conditions (30). To obtain ^57^Fe-enriched cells for Mössbauer spectroscopic analysis, iron was removed from glassware by three washing steps with 25% (wt/vol) HCl, followed by three rinses with 1/2-volume deionized water. MSR-1 cells were then cultivated in FSM supplemented with 40 μM ^57^Fe(citrate)_2_ (31).

10.1128/mBio.02795-18.1 TEXT S1 Supplemental methods used in this study. Download Text S1, DOCX file, 0.01 MB. Copyright © 2019 Uebe et al. 2019 Uebe et al. This content is distributed under the terms of the Creative Commons Attribution 4.0 International license.

### Molecular and genetic techniques.

Unless specified otherwise, molecular techniques were performed using standard protocols (32). All oligonucleotide primers (Text S1) were purchased from Sigma-Aldrich.

Point mutations were generated using the Phusion site-directed mutagenesis kit (Thermo Scientific).

### Generation of MSR-1 mutants.

For the generation of unmarked MSR-1 *dps* and *bfr* deletion mutants, ∼0.7-kb fragments of the up- and downstream regions of *dps* and *bfr12* were amplified by PCR using Phusion polymerase (New England BioLabs [NEB]) (primers [Text S1]). After gel purification of PCR products, the corresponding up- and downstream fragments were fused by overlap extension PCR with T4 polynucleotide kinase (Thermo Scientific) phosphorylated primers. The fused PCR products were subsequently cloned into dephosphorylated EcoRV-digested pORFM-GalK vector to yield pORFM*Δdps* and pORFM*Δbfr12*, respectively. Deletion vectors were transferred to MSR-1 by conjugation as described previously (33). Kanamycin-resistant plasmid insertion mutants were isolated after incubation for 5 days (30°C, 1.5% O_2_), transferred to 100 μl fresh FSM, and grown overnight (30°C, 1.5% O_2_). One-hundred-microliter cultures were then inoculated into 900 μl fresh FSM and incubated for 24 h at 30°C and 1.5% O_2_ before 100 μl cell culture was plated on FSM agar plates containing 2.5% galactose and 100 ng μl^−1^ anhydrotetracycline (34). After incubation for 5 days (30°C, 1.5% O_2_), mutant colonies were PCR verified and transferred to 100 μl FSM. For deletion of *bfr12* in the *Δdps* mutant, counterselection on FSM galactose plates was performed by incubation for 14 days under anaerobic conditions at ambient temperature using a Coy anaerobic chamber.

### Oxidative stress sensitivity assay.

To test growth of MSR-1 in the presence of O_2_ and/or H_2_O_2_, the WT, mutant, and pBam1-transcomplemented mutant strains (Table S1) were passaged three times in 3 ml FSM (without antibiotics) overnight at 28°C with 2% O_2_. Cultures were then diluted to an OD_565_ of 0.1 in FSM, and 100 μl of diluted cultures was used to inoculate 900 μl FSM containing 10 μM H_2_O_2_ (Merck). Aerobic growth (21% O_2_) at 141 rpm and 28°C was monitored every 20 min at 565 nm with an Infinite 200pro microplate reader (Tecan) for 80 h.

10.1128/mBio.02795-18.6 TABLE S1 Strains, plasmids, and oligonucleotides used in this study. Download Table S1, DOCX file, 0.02 MB. Copyright © 2019 Uebe et al. 2019 Uebe et al. This content is distributed under the terms of the Creative Commons Attribution 4.0 International license.

### Cloning, expression, and purification of recombinant MSR-1 Bfr.

Bfr from MSR-1 was overexpressed from pET-15b-Bfr in E. coli BL21-Gold cells and purified by SEC and AEC. Additionally, Bfr1, Bfr2, and Bfr12 were produced from pET-51b-Bfr1, pET-51b-Bfr2, and pET-51b-Bfr12 in E. coli Rosetta(DE3)pLysS, respectively (detailed methods for plasmid construction, gene expression, and protein purification are provided in Text S1).

### Determination of Bfr heme content.

Heme contents were determined by a pyridine hemochrome assay (35). Reduced minus oxidized difference spectra were recorded to identify heme groups. Heme concentrations were calculated applying a differential extinction coefficient, ε_557–540nm_ = 22.1 M^−1^ cm^−1^ (35).

### N-terminal Edman sequencing.

Purified Bfr12 was electroblotted on a PVDF membrane and stained with freshly prepared 0.1% Coomassie R-250 (in 40% methanol) and 1% acetic acid for 30 s. Destaining was performed with 50% methanol until the background was clear and bands were visible. The membrane was air dried, bands were cut, and N-terminal Bfr subunit amino acid sequences were determined by Edman degradation.

### BN-PAGE and 2D-BN/SDS-PAGE analyses.

To separate proteins by their native MW, we used 6 to 12% or 6 to 15% polyacrylamide gradient gels for BN-PAGE analyses as described previously (36). Therefore, Bfr12 samples were dialyzed against 50 mM imidazole-HCl, 50 mM NaCl, pH 7, at 4°C overnight (Visking type 8/32; MW cutoff [MWCO], 14 kDa [Carl Roth]). Alternatively, Bfr2 and Bfr1 plus -2 protein samples were adjusted to a concentration of 4.5 mg ml^−1^ in buffer A1 and diluted by addition of 50 mM Tris-HCl, pH 8.0, and 10× BN-PAGE loading buffer (50% [wt/vol] glycerol, 0.1% [wt/vol] Ponceau S) to a final concentration of 450 μg ml^−1^ (∼22 μM) protein, 5% (wt/vol) glycerol, and 0.01% (wt/vol) Ponceau S in 50 mM Tris-HCl, 50 mM NaCl, pH 8.0. Bfr1 samples were adjusted to a concentration of 6 mg ml^−1^ in buffer A1 and then diluted to a final concentration of 600 μg ml^−1^ (∼30 μM) protein, 5% (wt/vol) glycerol, and 0.01% (wt/vol) Ponceau S in 50 mM Tris-HCl, 50 mM NaCl, pH 8.0. Of these samples, 30 μl was loaded onto BN-PAGE gels with 10 μl of native marker liquid mix (Serva) as a control. The gels were then run at 4°C, 100 V, and 15 mA. After the run front reached one-third of the total run, the cathode buffer B (7.5 mM imidazole, 50 mM Tricine, 0.02% [wt/vol] Coomassie brilliant blue G-250) was replaced with cathode buffer B/10 (7.5 mM imidazole, 50 mM Tricine, 0.002% [wt/vol] Coomassie brilliant blue G-250), and the run was continued until the run front reached the end of the gel. BN-PAGE gels were then stained with Coomassie brilliant blue R-250 or subjected to 2D-BN/SDS-PAGE analysis.

For 2D-BN/SDS-PAGE, lanes of interest were cut out from BN-PAGE gels, briefly heated in 1% (wt/vol) SDS with 10 mM DTT, and equilibrated for 20 min. Treated gel strips were then clamped between glass plates of a gel casting apparatus, and an 8 to 22.5% gradient separation gel was poured underneath the gel strips. After polymerization, a 5% stacking gel was cast below the gel strip. After 4 μl PageRuler prestained protein ladder (Thermo Scientific) and 1 μg SDS-treated sample of the same protein from the first dimension were loaded, the SDS-PAGE gels were run at 25 mA per gel and 300 V for 3 h at room temperature (RT). Subsequently, gels were stained with Coomassie brilliant blue R-250.

### Determination of ferroxidase activities.

For the measurement of the ferroxidase activity, 1 μM Bfr was incubated with 150 μM FeCl_2_ in a total volume of 200 μl at 27°C, and the absorbance at 310 nm was measured over a period of 200 s (Evolution 201 UV-Vis spectrophotometer; Thermo Scientific). For this purpose, 178 μl of 50 mM MOPS, 50 mM NaCl, pH 7, mixed with 20 μl of 10 μM respective Bfr was used as a reference, and the reaction was started by the addition of 2 μl of 15 mM FeCl_2_ solution. As a negative control, 20 μl of buffer A1 was added instead of Bfr.

### Transmission Mössbauer spectroscopy.

For TMS analyses, MSR-1 WT, *ΔmamM*, and *Δbfr12* strains were grown in ^57^Fe-enriched medium and harvested at late exponential phase. Cell pellets were weighed, transferred into Delrin Mössbauer sample holders, frozen in liquid nitrogen, and kept at this temperature and in part at −80°C until measurement except for overnight transport on dry ice. The Mössbauer spectra were recorded in the horizontal transmission geometry using a constant acceleration spectrometer operated in conjunction with a 512-channel analyzer in the time scale mode. The detector consisted of a proportional counter filled with argon-methane (9:1). The source was at room temperature and consisted of 0.7 to 0.2 GBq [^57^Co] diffused in Rh foil (WissEl). The spectrometer was calibrated against α-iron at room temperature (RT). For measurements at 130 and 77 K, samples were placed in a continuous-flow cryostat (Oxford Instruments). For measurements at 4.2 K, a helium bath cryostat (MD306; Oxford Instruments) was employed. The ^57^Co source exhibiting an activity of 0.19 GBq was sealed in an Rh matrix at RT and was mounted on a constant-velocity drive. The detector consisted of a proportional counter made in-house and filled with argon-methane (90:10). Spectral data were buffered in a multichannel analyzer and transferred to a PC for further analysis, employing the Vinda program on an Excel 2003 platform (37). Isomer shifts δ, quadrupole splittings Δ*E_Q_* and Bhf, and percentages of the total absorption area were obtained by least-squares fits of Lorentzian lines to the experimental spectra.

### Transmission electron microscopy.

For TEM analysis, unstained cells were concentrated, adsorbed onto carbon- or Pioloform-coated copper grids, and washed two times with ddH_2_O (38). For TEM of Bfr and horse spleen ferritin samples, protein solutions were adsorbed on a carbon-coated copper grid for 10 min. Then, the grid was incubated for 3 min on uranyl acetate (2% [wt/vol]) for negative contrast and washed with ddH_2_O once. Bright-field TEM was performed on an FEI Tecnai F20 (FEI) or Zeiss EM902A (Zeiss) transmission electron microscope using an accelerating voltage of 200 kV or 80 kV, respectively. For data processing, interpretation, and analysis, the software packages DigitalMicrograph (Gatan) and ImageJ (NIH) were used. For determinations of magnetite particle numbers per cell, at least 100 cells were analyzed and at least 450 particles were measured for analysis of magnetite particle diameters. For determination of Bfr and ferritin diameters, at least 100 particles were analyzed.
