# Supplementary figures and images for: Bacterioferritin of Magnetospirillum gryphiswaldense Is a Heterotetraeicosameric Complex Composed of Functionally Distinct Subunits but Is Not Involved in Magnetite Biomineralization
Source: mBio. 2019 May 21;10(3):e02795-18. doi: 10.1128/mBio.02795-18 (PMC6529640; doi:10.1128/mBio.02795-18)

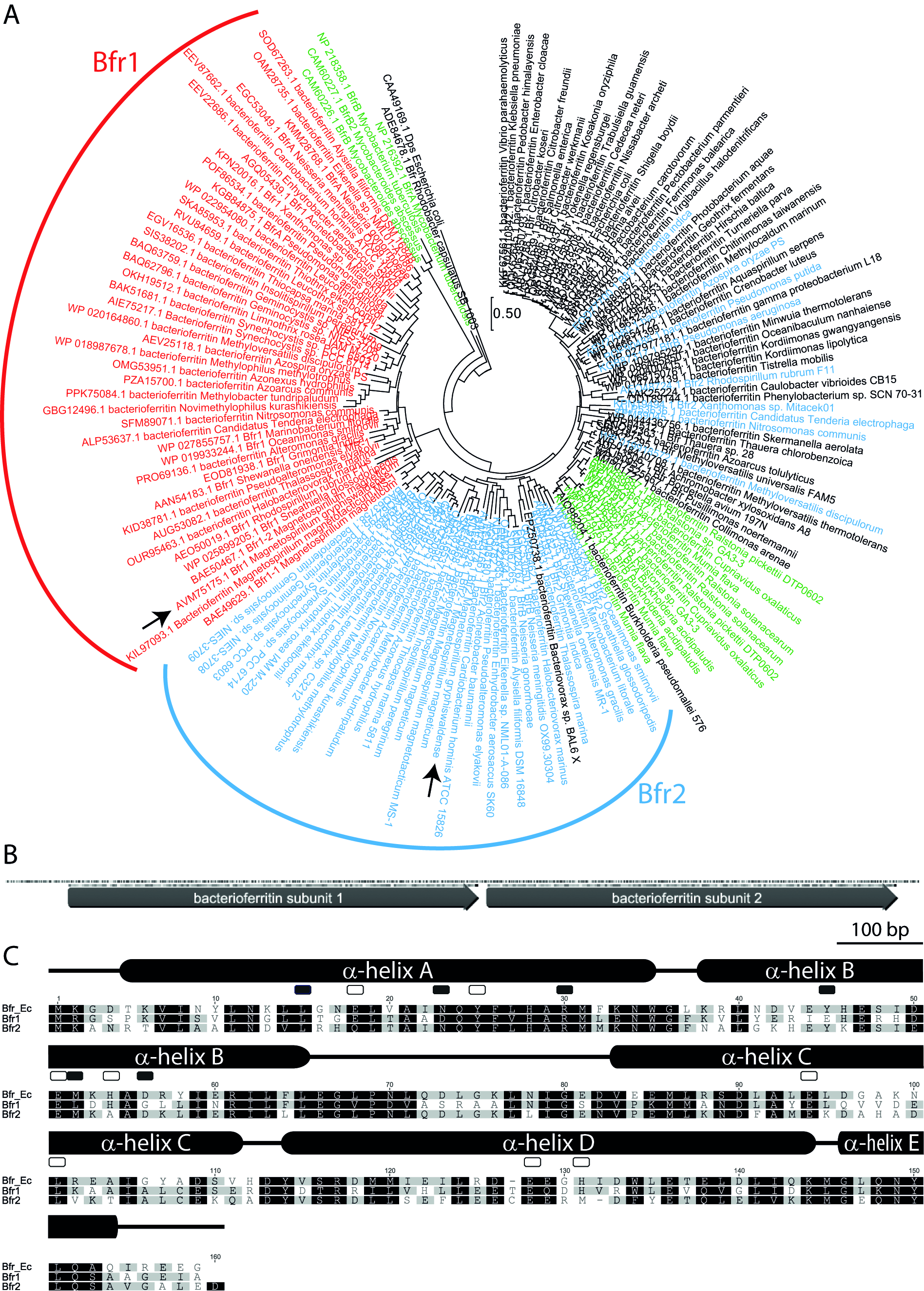

Supplement: FIG S1 [file mBio.02795-18-sf001.tif]

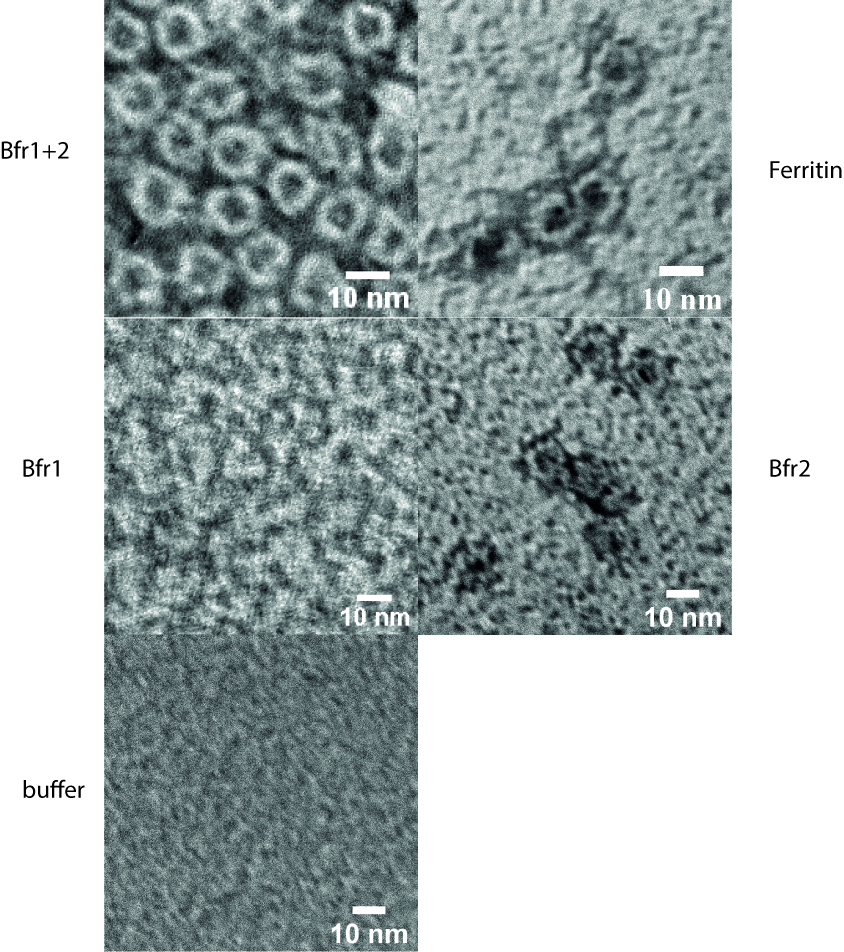

Supplement: FIG S2 [file mBio.02795-18-sf002.tif]

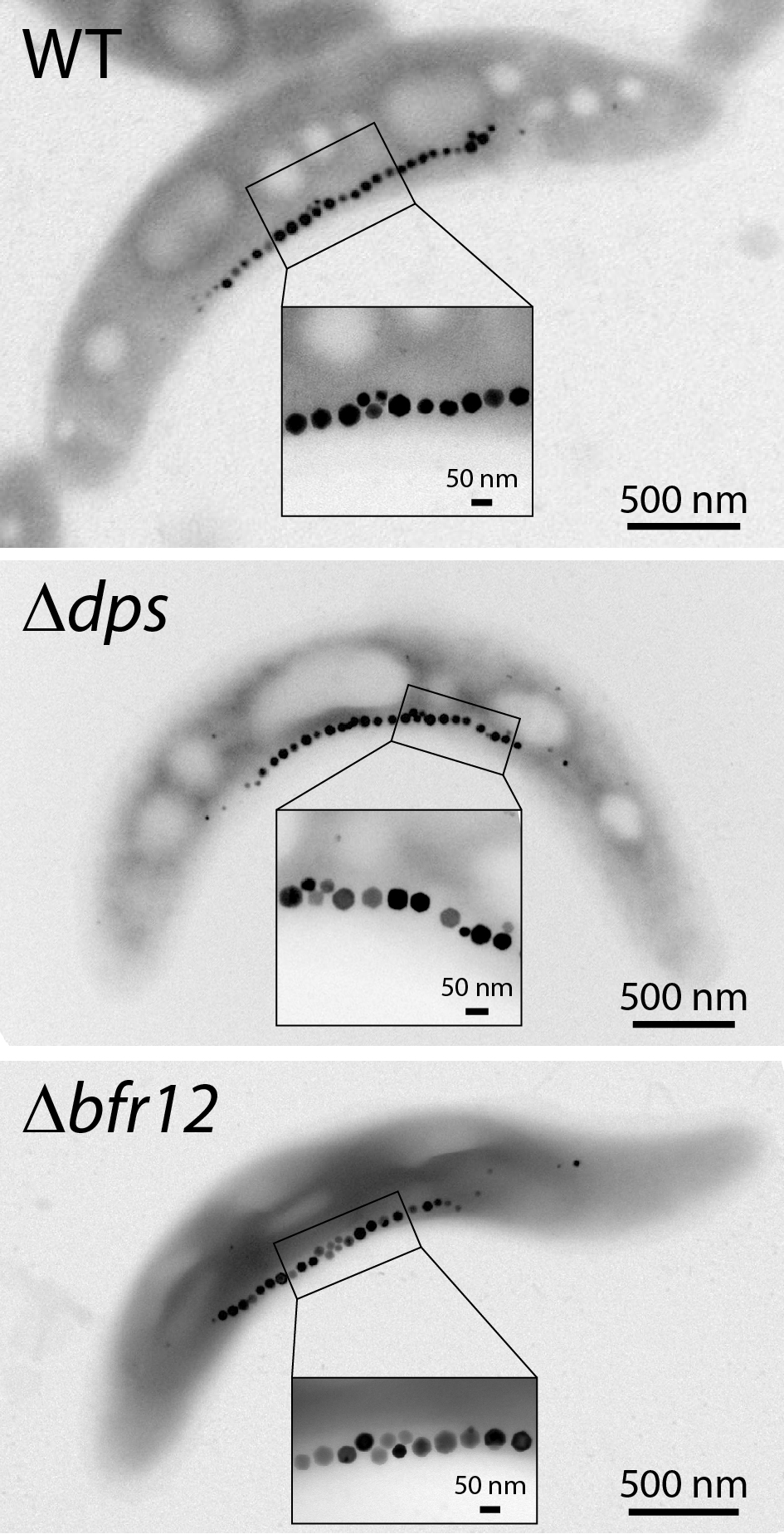

Supplement: FIG S3 [file mBio.02795-18-sf003.tif]

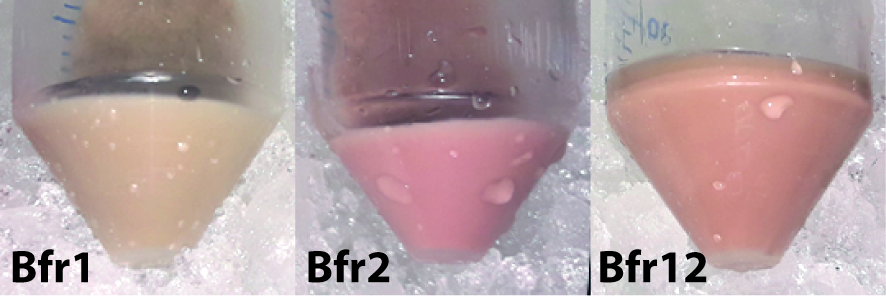

Supplement: FIG S4 [file mBio.02795-18-sf004.tif]
