# Supplementary material for: Bacterioferritin of Magnetospirillum gryphiswaldense Is a Heterotetraeicosameric Complex Composed of Functionally Distinct Subunits but Is Not Involved in Magnetite Biomineralization
Source: mBio. 2019 May 21;10(3):e02795-18. doi: 10.1128/mBio.02795-18 (PMC6529640; doi:10.1128/mBio.02795-18)
Supplement: TEXT S1 [file mBio.02795-18-s0001.docx]

**Supplemental methods**

**Cloning, expression and purification of the Bfr1 subunit using His-Tag**

The *bfr1* Gene was amplified from genomic DNA via PCR employing Pfu-Polymerase with primers: P3fwNde_f and P4rcBamHI_r containing cleavage sites for NdeI and BamHI. The gene was ligated into NdeI and BamHI digested pET15b vector and transformed into *E. coli* BL21-Gold. Overexpression was performed as described above, except that cells were harvested 5 hours after induction. Cells were lysed as described above. After cell lysis, cell debris was pelleted and the remaining superna­tant was centrifuged at 12,000 xg for 20 min at 4°C. The supernatant was dis­carded and the pellet was resuspended in 50 mM Hepes buffer, pH 7.5 with 1% Triton X-100. The pellet was centrifuged again (12,000 xg for 20 min at 4°C) and resuspended in 50 mM Hepes buffer, pH 7.5 with 1% Triton X-100 and 1 M NaCl. The suspension was centri­fuged (12,000 xg for 20 min at 4°C) and the pellet containing the inclusion bodies was washed in 50 mM Hepes buffer (pH 7.5). Inclusion bodies were solubilized in 8 M urea con­taining 0.5 M NaCl, 20 mM sodium phosphate, and 30 mM imidazole at pH 7.5 (binding buffer) for 30 min at room temperature. The protein solution was centrifuged at 12,000 xg for 20 min at 20°C. The supernatant containing rBfr1-His, was applied on a NiNTA column (HisTrap HP, GE Healthcare Life Sciences) with 0.25 ml min^-1^. The column was rinsed with binding buffer at 1 ml min^-1^. Bfr1-His was eluted in a 30 ml linear gradient (30 – 500 mM imidazole) of binding and elution buffer (20 mM sodium phosphate, 500 mM imid­azole, 0.5 M NaCl, 8 M urea, pH 7.5). The rBfr1-His protein was dialyzed against 25 mM Tris-HCl pH 7.5 overnight at 4°C under constant stirring (Spectra/Por3, cutoff 3.5 kDa, Spectrum Laboratories). After dialysis, the protein solution was centrifuged (12,000 xg, 4°C, 20 min). Su­pernatant and pellet were analyzed by SDS-PAGE and final protein concentration was deter­mined using a Pierce BCA protein assay kit (Thermo Scientific).

**Supplemental References**

1. **Marchler-Bauer A, Derbyshire MK, Gonzales NR, Lu S, Chitsaz F, Geer LY, Geer RC, He J, Gwadz M, Hurwitz DI, Lanczycki CJ, Lu F, Marchler GH, Song JS, Thanki N, Wang Z, Yamashita RA, Zhang D, Zheng C, Bryant SH.** 2015. CDD: NCBI's conserved domain database. Nucleic Acids Res **43**(Database issue)**:**D222-6. doi:10.1093/nar/gku1221.

2. **Schultheiss D, Schüler D.** 2003. Development of a genetic system for *Magnetospirillum gryphiswaldense.* Arch Microbiol **179**(2)**:**89–94. doi:10.1007/s00203-002-0498-z.

3. **Uebe R, Junge K, Henn V, Poxleitner G, Katzmann E, Plitzko JM, Zarivach R, Kasama T, Wanner G, Pósfai M, Böttger L, Matzanke B, Schüler D.** 2011. The cation diffusion facilitator proteins MamB and MamM of *Magnetospirillum gryphiswaldense* have distinct and complex functions, and are involved in magnetite biomineralization and magnetosome membrane assembly. Mol Microbiol **82**(4)**:**818–835. doi:10.1111/j.1365-2958.2011.07863.x.

4. **Raschdorf O, Plitzko JM, Schüler D, Müller FD.** 2014. A tailored *galK* counterselection system for efficient markerless gene deletion and chromosomal tagging in *Magnetospirillum gryphiswaldense.* Appl Environ Microbiol **80**(14)**:**4323–4330. doi:10.1128/AEM.00588-14.

5. **Martínez-García E, Calles B, Arévalo-Rodríguez M, Lorenzo V de.** 2011. pBAM1: an all-synthetic genetic tool for analysis and construction of complex bacterial phenotypes. BMC Microbiol **11**(1)**:**38. doi:10.1186/1471-2180-11-38.

6. **Karimova G, Dautin N, Ladant D.** 2005. Interaction network among *Escherichia coli* membrane proteins involved in cell division as revealed by bacterial two-hybrid analysis. J Bacteriol **187**(7)**:**2233–2243. doi:10.1128/JB.187.7.2233-2243.2005.
