# Supplementary material for: Bacterioferritin of Magnetospirillum gryphiswaldense Is a Heterotetraeicosameric Complex Composed of Functionally Distinct Subunits but Is Not Involved in Magnetite Biomineralization
Source: mBio. 2019 May 21;10(3):e02795-18. doi: 10.1128/mBio.02795-18 (PMC6529640; doi:10.1128/mBio.02795-18)
Supplement: TABLE S1 [file mBio.02795-18-st001.docx]

**Table S1.** Strains, plasmids and oligos used in this study.

| Strain | Important features | Source or reference |
| --- | --- | --- |
| ***E. coli*** |  |  |
| DH5α | F' Φ80d*lac* ∆M15 ∆(*lacZYA*-*argF*)U169 *deo*R *rec*A1 *end*A1 | Invitrogen |
| BW29427 | *thrB1004 pro thi rpsL hsdS lacZ*∆M15 RP4-1360 ∆(*araBAD*)567 ∆*dapA1341::[erm pir* (wt)*]* | Datsenko, K. and Wanner B. L. (unpub.) |
| BL21 Gold | F^−^ ompT hsdS(r_B_^−^ m_B_^−^) dcm^+^ Tet^R^ galλ(DE3) endA Hte | Stratagene |
| XL-1 Blue  Rosetta (DE3) pLys | recA1 endA1 gyrA96 thi-1 hsdR17 supE44 relA1 lac [F′ proAB lacI^q^ZΔM15] Tn10 (Tet^r^)  F^-^ *ompT hsdS_B_*(r_B_^−^ m_B_^−^) *gal dcm* (DE3) pLysSRARE (Cam^R^) | Stratagene  Novagen |
|  |  |  |
| ***M. gryphiswaldense*** |  |  |
| MSR-1 R3/S1 | Rif^r^, Sm^r^ spontaneous mutant, wild type | (3) |
| ∆*mamM* | R3/S1 but ∆*mamM* | (4) |
| ∆*bfr12* | R3/S1 but ∆*bfr1 and* *∆bfr2* | this study |
| ∆*dps* | R3/S1 but ∆*dps* | this study |
| ∆*dps/*∆*bfr12* | R3/S1 but ∆*dps,* ∆*bfr1 and* *∆bfr2* | this study |
|  |  |  |
| Plasmid | Important features | Source or reference |
| pORFM-GalK | General backbone vector for GalK counterselection; *npt galK tetR mobRK2* | (5) |
| pORFM∆*bfr12* | pORFM GalK + ∆*bfr12* | this study |
| pORFM∆*dps* | pORFM GalK + ∆*dps* | this study |
| pBam1 | Km^R^, Ap^R^, oriR6K, *tnpA* | (6) |
| pBam1-*dps* | pBam1 with P*_mamG_* and *Dps* | this study |
| pBam1-*bfr12* | pBam1 with P*_mamG_* and *Bfr1+2* | this study |
| pBam1-*bfr1* | pBam1 with P*_mamG_* and *Bfr1* | this study |
| pBam1-*bfr2* | pBam1 with P*_mamG_* and *Bfr2* | this study |
| pUT18c | BACTH vector N-terminal T18 fragment (amino acids 225–399 of CyaA); ColE1 ori; Ap^R^ | (7) |
| pUT18c-*zip* | pUT18c derivative with GCN4 leucine zipper fused to T18 fragment; Ap^R^ | (7) |
| pUT18c-*bfr1* | *bfr1* inserted in XbaI and EcoRI sites of pUT18c | this study |
| pUT18c-*bfr2* | *bfr2* inserted in XbaI and EcoRI sites of pUT18c | this study |
| pKT25-*zip* | pKT25 derivative with GCN4 leucine zipper fused to T25 fragment; Km^R^ | (7) |
| pKNT25 | BACTH vector, C-terminal T25 fragment (amino acids 1–224 of CyaA); p15 ori; Km^R^ | (7) |
| pKNT25-*bfr1* | *bfr1* inserted in XbaI and EcoRI sites of pKNT25 | this study |
| pKNT25-*bfr2*  pET-15b  pET-15b-Bfr  pET-15b-Bfr1  pET-51b  pET-51b-Bfr12  pET-51b-Bfr1  pET-51b-Bfr2 | *Bfr2* inserted in XbaI and EcoRI sites of pKNT25  T7-Pol expression vector, N-terminal 6xHis-tag  *bfr12* inserted in NcoI and NdeI sites of pET-15b  *bfr1* inserted in NdeI and BamHI sites of pET-15b  T7-Pol expression vector, N-terminal StrepII-tag, C-terminal 10xHis-tag  *bfr12* inserted in KpnI and SacI sites of pET-51b  *bfr1* inserted in KpnI and SacI sites of pET-51b  *bfr1* inserted in KpnI and SacI sites of pET-51b | this study  Novagen  this study  this study  Novagen  this study  this study  this study |
| pET-51b-Bfr12ΔFC  pET-51b-Bfr12M52L | *bfr1* encoding FC mutations E18Q, H54A, H131M, *bfr2* wt  *bfr1* wt*, bfr2* encoding a M52L mutation | this study  this study  this study |
| Oligo | Sequence (5’→3’) |  |
| Dps_del_do_for | CCGCCCTTTTCGTAGATCATCTCCTTTCACGGGTTG | |
| Dps_del_do_rev | ATATGCATGGTCTCGATTTCCGGC | |
| Dps_del_up_for | AGCAATGGCAACGAAAAAGACGACG | |
| Dps_del_up_rev | GAAAGGAGATGATCTACGAAAAGGGCGGGGCGCTTG | |
| Bfr_del_do_for | TTAAGGAGATGAATAGTTTTTGACCTCCGGCGC | |
| Bfr_del_do_rev | TGTGCGGGTCAGCCAGTTTT | |
| Bfr_del_up_for | GGCAGGGCCATCGACATCAT | |
| Bfr_del_up_rev | GGAGGTCAAAAACTATTCATCTCCTTAAGTAAC | |
| Bfr1_KpnI_for | GGTACCATGCGCGGAAGCCCGAAAGT | |
| Bfr1_SacI_rev | GAGCTCTTACGCGATCTCGCCAGCGG | |
| Bfr2_KpnI_for | GGTACCATGAAAGCCAACCGCACCGT | |
| Bfr2_SacI_rev | GAGCTCTCAGTCCTCCAAGGCGCCGA | |
| Dps_KpnI_for | GGTACCATGGCCAAGAACAAAGCCGCTAAAATCGAG | |
| Dps_SacI_rev | GAGCTCTCAGCCCAGCAGGGCGCGCA | |
| Bfr1_for_XbaI | TCTAGAGCGCGGAAGCCCGAAAGTGAT | |
| Bfr1_rev_EcoRI | GAATTCTCCGCGATCTCGCCAGCGGCGC | |
| Bfr2_for_XbaI | TCTAGAGAAAGCCAACCGCACCGTGCTGGC | |
| Bfr2_rev_EcoRI | GAATTCTCGTCCTCCAAGGCGCCGACGG | |
| Dps_for_XbaI | TCTAGAGGCCAAGAACAAAGCCGCTAA | |
| Dps_rev_EcoRI | GAATTCTCGCCCAGCAGGGCGCGCAG | |
| P1_f | GTATGCGCGGAAGCCCGAAA | |
| P2_r | ACTTCAGTCCTCCAAGGCG | |
| P5fwBspHI_f | GGTCATGAGGGGAAGCCCGAAA | |
| P6revNdeI_r | GAGAACCCCATATGACTTCAGTCCT | |
| P3fwNde_f | GAGATGCATATGCGCGGAAGC | |
| P4rcBamHI_r  Bfr1_KpnI_for  Bfr1_SacI_rev  Bfr2_KpnI_for | ACGGATCCTTACGCGATCTCG  GCATCAGGTACCGCGCGGAAGCCCGAAAGTGATAAGC  GAGCTCTTACGCGATCTCGCCAGCGG  GGTACCATGAAAGCCAACCGCACCGT | |
| Bfr2_SacI_rev | GCTTCGAGCTCTTATCAGTCCTCCAAGGCGCCGAC | |
| Bfr1_E18Q_for  Bfr1_E18Q_rev  Bfr1_H54A_for  Bfr1_H54A_rev  Bfr1_H131M_for  Bfr1_H131M_rev  Bfr2_M52L_for  Bfr2_M52L_rev | ACGGGGCAGCTGACCGCTGC  CAGCAGCCCATTGAGAACGCTTATCAC  TTGGACGCTGCCGGCTTGCTG  CTCGTCATGGCGTTCATGCTCGATG  CAGGACATGGTGCGCTGGCTGG  CTCGGTCTCTTCCAGCAGATGGACCAG  GAGGAGTTGAAGGCCGCCGACAAG  GATGGATTCCTTGTATTCATGCTTACCCAGG | |
